# The Story of the Insane from Year to Year

**Published:** 1903-02-07

**Authors:** 


### THE STORY OF THE INSANE FROM YEAR TO YEAR.

#### Fifteenth Annual Series.

### LONDON COUNTY ASYLUM AT BANSTEAD.

Here 2,436 patients were in residence on January 1st, and by December 31st the numbers had increased to 2,457. There were 435 first admissions, 87 readmissions, 194 discharged recovered, 21 discharged relieved, 105 discharged not improved, and 181 died. The average number resident was 2,442, and the total number under care during the year was 2,958. The recoveries were 39·67 per cent. of the admissions, and the deaths were 7·41 per cent. of the average number resident. Of the year's deaths 86 were caused by cerebral and spinal diseases, general paralysis alone accounting for 55 of this number, consumption caused death in 32, and other lung diseases in 22. Of the year's admissions 75 owed their insanity to various moral causes. The chief physical cause was intemperance in drink, which was responsible for 93, while hereditary influence accounted for 57. Dr. D. Johnston Jones succeeded Dr. Claye Shaw as medical superintendent, the latter having retired on his pension. Pensions were also granted to Mr. Hosegood the steward, to Miss Wanstall the assistant matron, to J. B. Randall the baker, to Charge-attendant Whitfield, to Nurse Petherick, and to F. Longhirst. The committee say they "have satisfaction in recording that under Dr. Johnston Jones assisted by the medical and general staff, the well-being of the patients and the condition of the asylum generally are efficiently maintained." The weekly cost per head was a fraction under 10s. 8d.

### THE ROYAL ASYLUM, PERTH.

At the beginning of the year there were on the books the names of 132 patients, and at the end of it the numbers had risen to 143. There were 34 first admissions, 4 readmissions, 17 discharged recovered, 10 discharged relieved, and 5 died. The average number resident during the year was 127, and the total number under care was 196. The percentage of recoveries on admissions is very creditable, as it stands at 44·73, and that of the deaths, calculated on the average number resident, is only 3·13. Of the admissions, 24 were hereditarily predisposed to insanity, and 3 others belonged to families having neurotic tendencies. When analysing the admissions, and referring to the drink cause, Dr. Urquhart finds that only 12 per cent. of his cases were caused by alcohol, and he points out that this is only about half of what is found in county asylums. "It shows," he says, "in dealing with patients drawn exclusively from the middle class, that the number suffering from the serious results of alcoholism is less than in asylums receiving the industrial classes—not to the full extent indicated by figures perhaps, but in any case such a diminution as may lead us to expect that as drunkenness has declined in frequency amongst the educated, so it will decline in all ranks of society." We are the children of Hope, so let us pick up a grain of comfort when we can. The asylum is now full; but a villa is being put up for those patients who can afford the higher rates of board. The total revenue for the year was £13,615, and the total expenditure was £12,112. The average board rate is £88 19s. per annum. Patients from Perthshire pay from £30 to £52, and out-county cases from £84. The Commissioners say that "the administration of the asylum continues to be characterised by great ability and thoughtfulness. The patients are provided for in a liberal manner, and their individual requirements evidently receive careful consideration."

